# “Efficacy of SGLT2 inhibitors in non-diabetic non-alcoholic fatty liver disease: a systematic review and meta-analysis”

**DOI:** 10.1007/s40200-025-01797-0

**Published:** 2025-12-04

**Authors:** Muhammad Sharjeel Abbas, Mrunalini Dandamudi, Tooba Rehman, Muhammad Aqib Faizan, Izza Zahra, John Cedric Mojica, Musab Riyan Ahmed, Karishma Bai, Izza Shakeel, Zunaira Shahzad, Juliana Giorgi

**Affiliations:** 1 Gomal Medical College, Khyber Medical University, Peshawar, Pakistan; 2 Montefiore Medical Center, Moses Campus, Bronx, New York, USA; 3 Foundation University Medical College, Islamabad, Pakistan; 4 University of the East Ramon Magsaysay Medical Center, Quezon City, Metro Manila, Philippines; 5 Department of Medicine, Allama Iqbal Medical College, Lahore, Punjab, Pakistan; 6 People’s University of Medical and Health Sciences for Women (PUMHS), Nawabshah, Sindh, Pakistan; 7 Fatima Jinnah Medical University, Lahore, Punjab, Pakistan; 8 Sirio Libanes Hospital, São Paulo, Brazil; 9 Albert Einstein Hospital, São Paulo, Brazil

## Abstract

**Background:**

Non-alcoholic fatty liver disease (NAFLD), now termed metabolic dysfunction-associated steatotic liver disease (MASLD), is a common chronic liver condition with significant metabolic and cardiovascular implications. Although sodium-glucose cotransporter 2 inhibitors (SGLT2i) have demonstrated hepatic benefits in diabetic populations, their role in non-diabetic individuals with NAFLD remains unclear.

**Objective:**

This meta-analysis aimed to evaluate the effects of SGLT2 inhibitors in non-diabetic NAFLD/MASLD patients.

**Methods:**

PubMed, Embase, and CENTRAL were searched up to May 2025 for randomized controlled trials (RCTs) comparing SGLT2i with placebo or other pharmacologic agents in non-diabetic adults with NAFLD. Primary outcomes included changes in hepatic function; secondary outcomes assessed anthropometric, metabolic, and imaging-based markers of hepatic steatosis and fibrosis. A random-effects model was applied to estimate pooled mean differences (MDs) and 95% confidence intervals (CIs).

**Results:**

Five RCTs comprising 273 non-diabetic patients (142 in the SGLT2i group) were included. SGLT2i significantly improved liver enzymes: AST (MD = -2.03; 95% CI: -3.24 to -0.82; *p* < 0.01), ALT (MD = -4.50; 95% CI: -6.89 to -2.10; *p* < 0.01), and GGT (MD = -4.12; 95% CI: -6.87 to -1.37; *p* < 0.01). Modest but significant reductions were also observed in body weight (MD = -3.24 kg), BMI (MD = -1.02 kg/m²), and waist circumference (MD = -3.12 cm). However, SGLT2i did not significantly affect triglycerides, HbA1c, fasting glucose, liver stiffness, CAP scores, or FIB-4 index.

**Conclusion:**

SGLT2i significantly improves liver enzyme levels and anthropometric markers in non-diabetic individuals with NAFLD/MASLD, suggesting potential therapeutic benefits. However, their effects on hepatic steatosis resolution and fibrosis progression remain inconclusive.

**Graphical Abstract:**

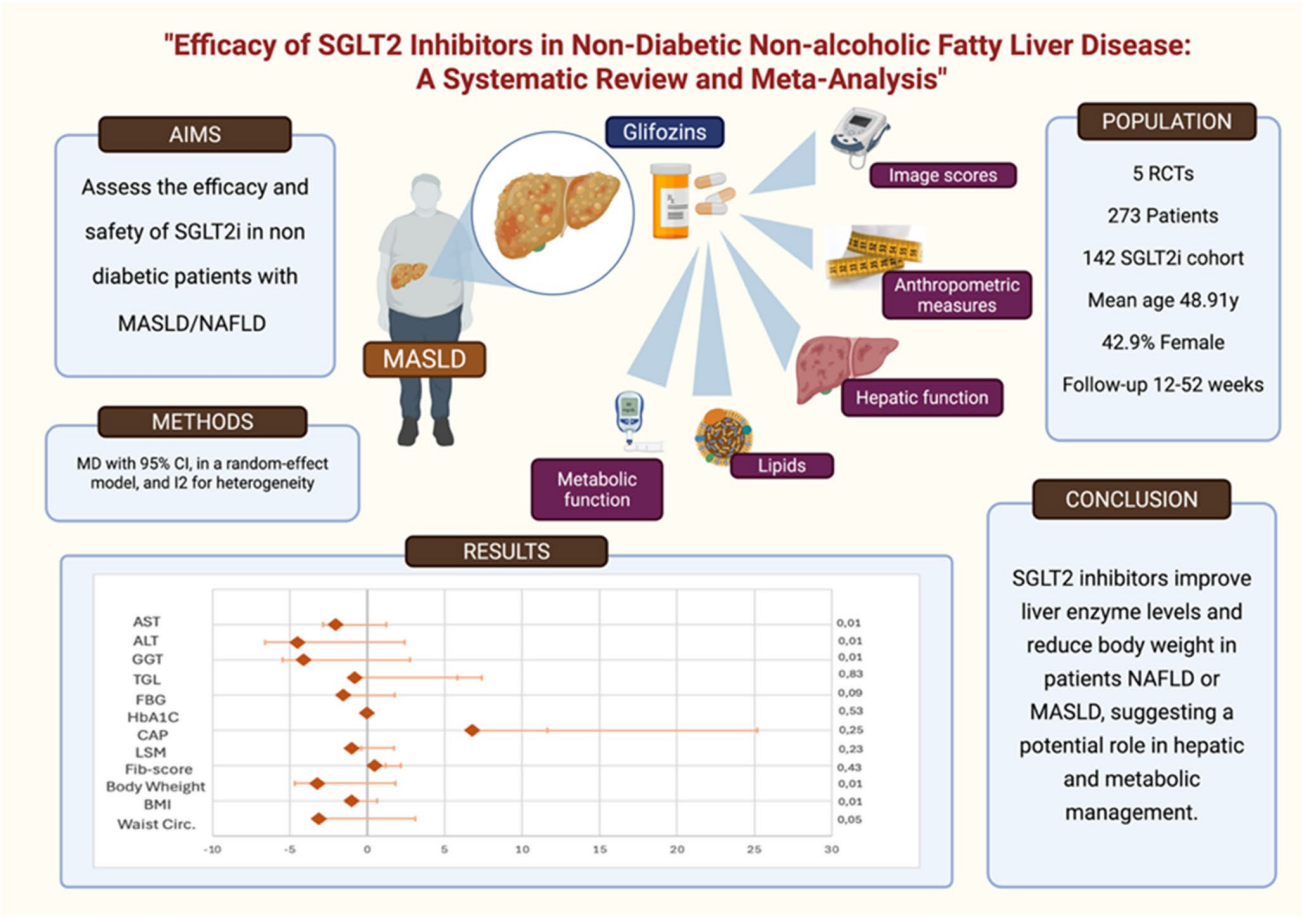

**Supplementary Information:**

The online version contains supplementary material available at 10.1007/s40200-025-01797-0.

## Introduction

Non-alcoholic fatty liver disease (NAFLD), recently known as metabolic dysfunction-associated steatotic liver disease (MASLD) [1], is the most prevalent, chronic liver disease, affecting approximately 25% of the general population [2–4]. It is defined as the excessive accumulation of fat (≥ 5%) in hepatocytes with little or no significant alcohol consumption [5, 6]. The spectrum of NAFLD ranges from a simple increase in hepatic lipid content (steatosis, non-alcoholic fatty liver) to non-alcoholic steatohepatitis (NASH), fibrosis, liver cirrhosis, and ultimately increased risk of hepatocellular carcinoma [7, 8]. The pathophysiology of NAFLD includes insulin resistance, oxidative stress, mitochondrial dysfunction, and lipotoxicity [9, 10]. NAFLD is usually accompanied by several complications such as Type 2 diabetes mellitus (T2DM), cardiovascular diseases (CVD), and chronic kidney disease (CKD) [11]. The leading cause of death in these patients is cardiovascular-related, not hepatic [12]. While NAFLD is more common among individuals with diabetes [13], it is increasingly being observed in non-diabetic populations as well [14].

According to recent guidelines, as per the American Association for the Study of Liver Diseases (AASLD) Guidance, vitamin E, pioglitazone, statins, and omega-3 fatty acids are recommended as therapeutic agents for NAFLD. The lifestyle modifications, dietary restrictions, and physical activity for weight reduction are also effective ways to improve hepatic fat content. Still, there are no FDA-approved drugs for the specific management of NAFLD patients with and without diabetes [15–17].

Sodium–glucose co-transporter 2 inhibitors (SGLT2i) are a class of oral medications primarily used to treat T2DM. They work by blocking the SGLT2 protein in the kidneys, which is responsible for reabsorbing glucose back into the blood. By inhibiting this protein, more glucose is excreted in the urine, thereby lowering blood sugar levels. SGLT2i are a class of oral antidiabetic medications that significantly reduce blood glycemic levels and increase urinary glucose excretion by inhibiting renal tubular glucose reabsorption [18]. SGLT2 inhibitors, such as empagliflozin and dapagliflozin, have demonstrated positive effects in NAFLD patients by reducing liver fat content and improving liver function tests in predominantly diabetic patients. However, the impact of SGLT2i on NAFLD patients without diabetes remain unclear and inconclusive.༈[19–21]༉.

The number of randomized controlled trials (RCTs) assessing the efficacy of SGLT2i in non-diabetic patients with NAFLD has been steadily growing. However, findings remain inconsistent, with variability in reported outcomes regarding hepatic fat reduction and liver function improvement. This meta-analysis aims to comprehensively evaluate the impact of SGLT2i compared to placebo or other pharmacological agents on hepatic fat content and liver biochemical markers in non-diabetic individuals with NAFLD.

## Methods

This systematic review and meta-analysis were conducted by the Preferred Reporting Items for Systematic Reviews and Meta-Analyses (PRISMA) 2020 statement and the Cochrane Collaboration Handbook for Systematic Reviews of Interventions [22].

### Eligibility criteria

We included studies that met the following criteria: (1) RCTs; (2) involved adult non-diabetic patients with biopsy-proven or imaging-based diagnosis of metabolic dysfunction-associated steatotic liver disease (MASLD) or non-alcoholic steatohepatitis (NASH); (3) compared SGLT2i (including dapagliflozin, canagliflozin, empagliflozin, luseogliflozin, or ertugliflozin) with placebo or other antidiabetic drug; and (4) reported at least one outcome of interest. The primary outcomes included a reduction in hepatic fat content and improvement in liver function tests. Secondary outcomes included changes in liver fibrosis scores, anthropometric measures, and metabolic biomarkers.

We excluded studies that: (4) included diabetic patients or mixed populations without subgroup analysis of non-diabetic patients; (2) evaluated supplements or agents other than SGLT2i; (3) were non-randomized studies, case reports, reviews, or conference abstracts; and (4) reported overlapping populations.

### Search strategy and data extraction

A comprehensive literature search was performed in PubMed, Embase, and the Cochrane Central Register of Controlled Trials (CENTRAL) from inception to May 2025 using the following keywords: (“Sodium-Glucose Transporter 2 Inhibitors” OR dapagliflozin OR empagliflozin OR canagliflozin OR ertugliflozin) AND (“Fatty Liver” OR NAFLD OR “nonalcoholic fatty liver disease” OR NASH OR “nonalcoholic steatohepatitis” OR “Metabolic dysfunction-associated steatotic liver disease” OR MASLD OR MAFLD) AND (nondiabetic OR non-diabetic OR “without diabetes”). Additional studies were identified by manually screening the references of included articles.

Two authors independently screened titles and abstracts for eligibility, with disagreements resolved through discussion. Full texts of potentially eligible studies were retrieved and reviewed against the inclusion and exclusion criteria. The study protocol was registered prospectively in PROSPERO ID: CRD420251107455.

## Quality assessment

The risk of bias for each included RCT was assessed using the Cochrane Risk-of-Bias 2.0 (RoB 2) tool [23], which evaluates five domains: randomization process, deviations from intended interventions, missing outcome data, measurement of outcomes, and selection of reported results. Two reviewers independently performed the assessment, and disagreements were resolved by consensus.

### Statistical analysis

For continuous outcomes, mean differences (MDs) with 95% confidence intervals (CI) were calculated. Mean differences were calculated using the inverse variance method with random-effects modeling (DerSimonian–Laird estimator), as recommended by the Cochrane Handbook [24] for Systematic Reviews of Interventions.

Heterogeneity was assessed using Cochran’s Q test and the I² statistic, with *p* < 0.10 and I² >25% considered significant. We adopted a conservative threshold (I² >25%) to indicate heterogeneity, aligning with Higgins et al. (2002), who classify 25–50% as moderate and >75% as considerable heterogeneity [25]. This criterion was used to ensure sensitivity between study variability given the small number of included RCTs. All analyses were conducted using Review Manager (RevMan) version 5.4.1.

Publication bias was assessed using Egger’s regression test for funnel plot asymmetry across all outcomes.

## Results

### Study selection and baseline characteristics

**Fig. 1:**
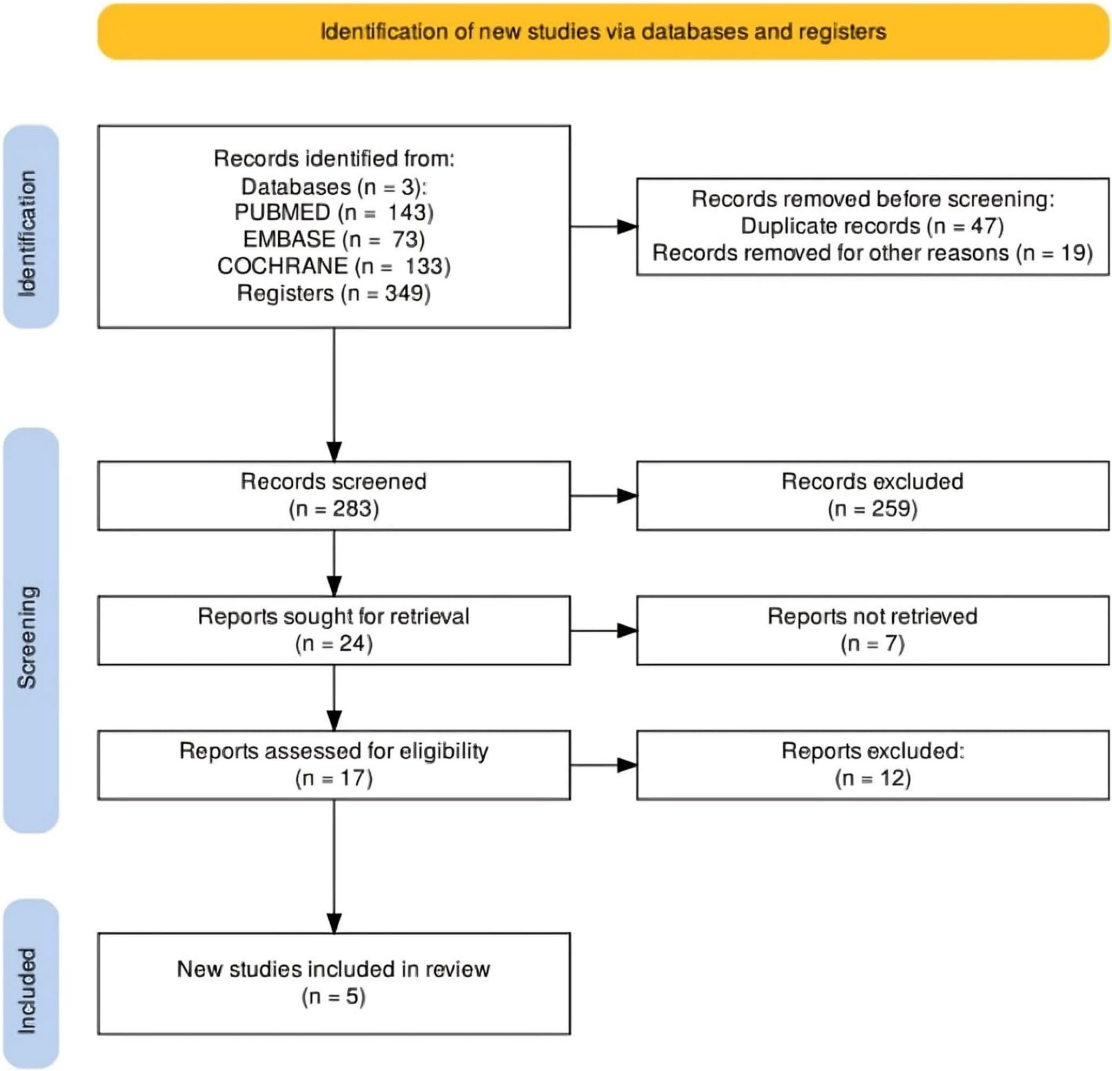
PRISMA flow diagram of study screening and selection

The initial search yielded 349 results. After removing duplicates and unrelated studies based on title and abstract, we retrieved 17 studies for full-text review (Fig. 1). Of those, 5 RCTs (*n* = 273 participants) met the inclusion criteria, involving a total of 273 non-diabetic patients with NASH: 142 in the SGLT2 inhibitors group and 131 in the control group, represented as placebo or another antidiabetic drug (pioglitazone or tenegliptin). The baseline characteristics of the studies are outlined in Table 1. The studies presented a median age of 48.9 years, with 42.9% of the participants being females.

**Table 1:** Baseline characteristics of included studies

Author	Country	Female, *n* (%) (SGLT2i vs. Control)		Sample Size	Age (years)	BMI (kg/m^2^)	Interventions (SGLT2i vs. Control)		ALT(U/L)	AST (U/L)	Fibrosis-4 Index	Follow-up (weeks)
Taheri 2020	Iran	15 (34.9)	25 (53.2)	90	44.0 ± 9.4	30.6 ± 3.0	Empagliflozin (10 mg/day)	Placebo	36.1 ± 22.2	25.3 ± 9.7	0.9 ± 0.4	24
Tobita 2020	Japan	4 (33.3)	3 (30.0)	22	47.2 ± 15.0	28.3 ± 4.3	Dapagliflozin (5 mg/day)	Teneligliptin (20 mg/day)	86.4 ± 38.8	49.4 ± 10.9	1.1 ± 0.4	12
Abdelgani 2024	USA	8 (44.0)	5 (55.0)	27	45.0 ± 3.3	30.5 ± 3.4	Empagliflozin (25 mg/day)	Placebo	33.7 ± 4.7	21.3 ± 2.2	0.7 ± 0.1	12
Abdel Monem 2025	Egypt	15 (60.0)	20 (80.0)	38	43.4 ± 9.4	37.5 ± 7.7	Dapagliflozin (10 mg/day)	Pioglitazone (30 mg/day)	51.0 ± 32.4	48.1 ± 22.4	1.3 ± 0.7	24
Cheung 2025	Hong Kong	22 (44.9)	22 (44.9)	98	55.9 ± 3.1	27.5 ± 3.5	Empagliflozin (10 mg/day)	Placebo	30.7 ± 16.1	25.7 ± 7.4	N/A	52

**Abbreviations** = BMI: Body Mass Index; kg/m^2^: kilogram per square meter; mg: milligram; SGLT2i: Sodium Glucose Cotransporter-2 Inhibitors; U/L: units per liter; USA: United States of America

### Primary outcomes

**Fig. 2:**
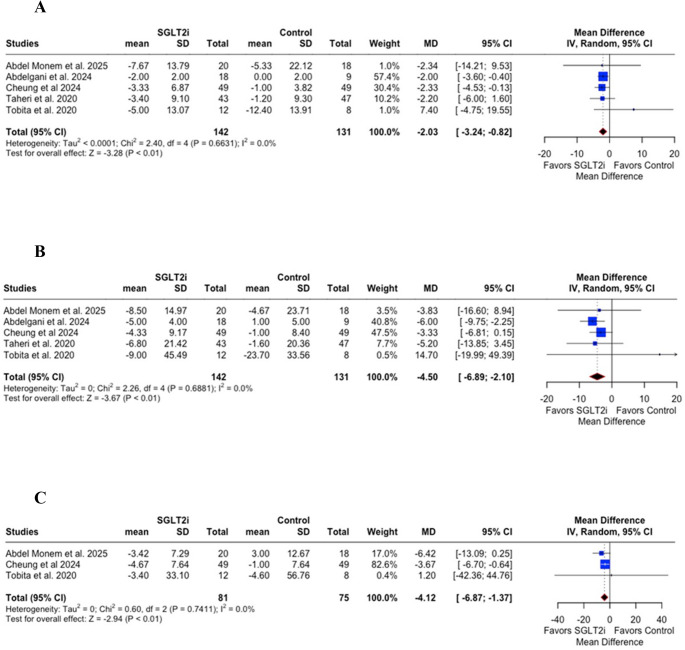
** A** – Aspartate aminotransferase (AST) levels were significantly reduced in the SGLT2i group compared with controls (*p* < 0.01), **B** – Alanine aminotransferase (ALT) levels were significantly reduced in the SGLT2i group compared with controls (*p* < 0.01), **C** – Gamma-glutamyl transferase (GGT) levels were significantly reduced in the SGLT2i group compared with controls (*p* < 0.01). CI = Confidence interval; MD = Mean difference

Treatment with SGLT2i resulted in significant improvements in liver enzymes. Aspartate aminotransferase (AST) levels decreased significantly with a mean difference (MD) of −2.03 (95% CI: −3.24 to −0.82; *p* < 0.01; I² = 0%; Fig. 2A). Alanine aminotransferase (ALT) was also significantly reduced (MD = −4.50; 95% CI: −6.89 to −2.10; *p* < 0.01; I² = 0%; Fig. 2B), as was gamma-glutamyl transferase (GGT) (MD = −4.12; 95% CI: −6.87 to −1.37; *p* < 0.01; I² = 0%; Fig. 2C).

### Secondary outcomes

**Fig. 3:**
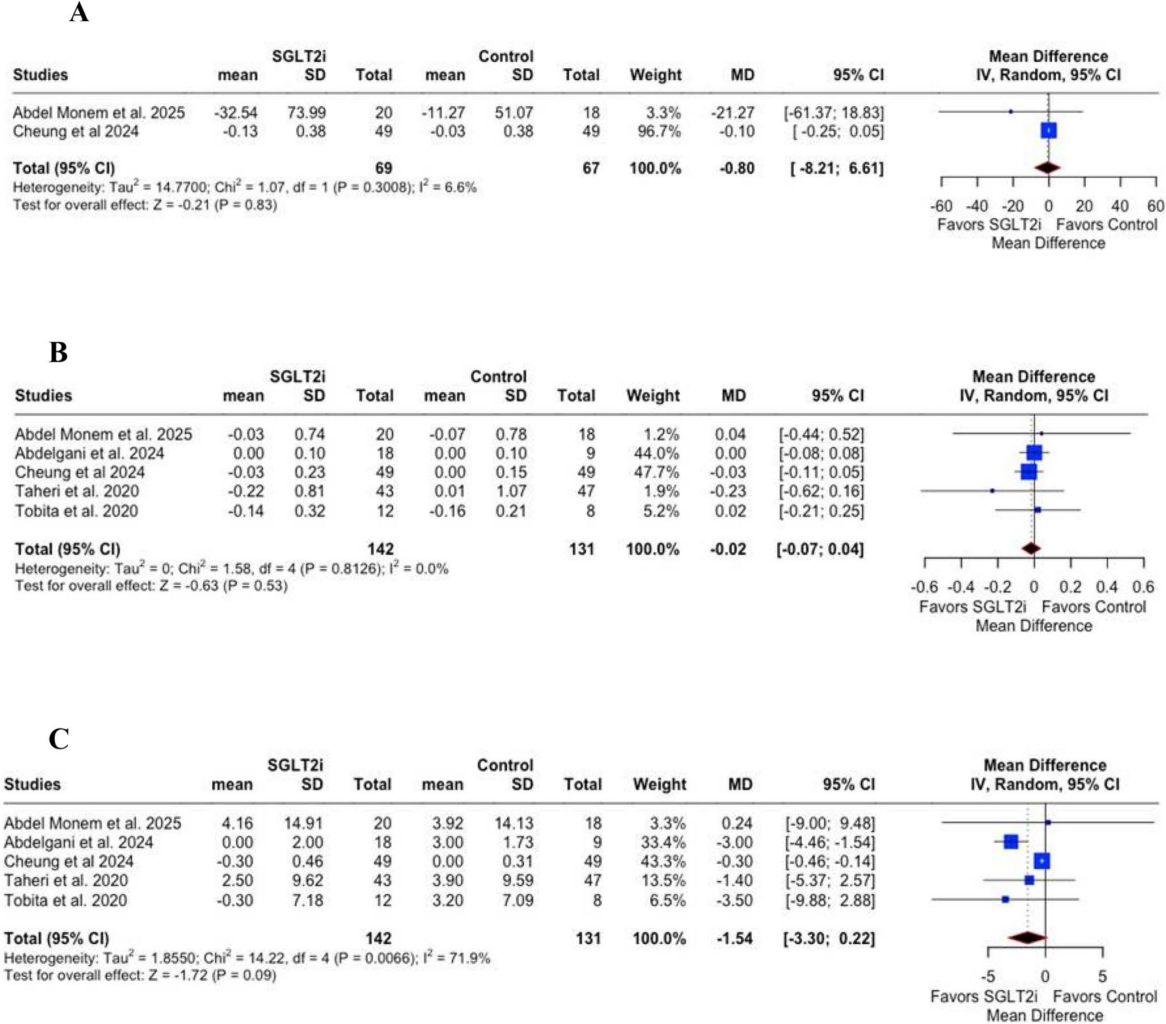

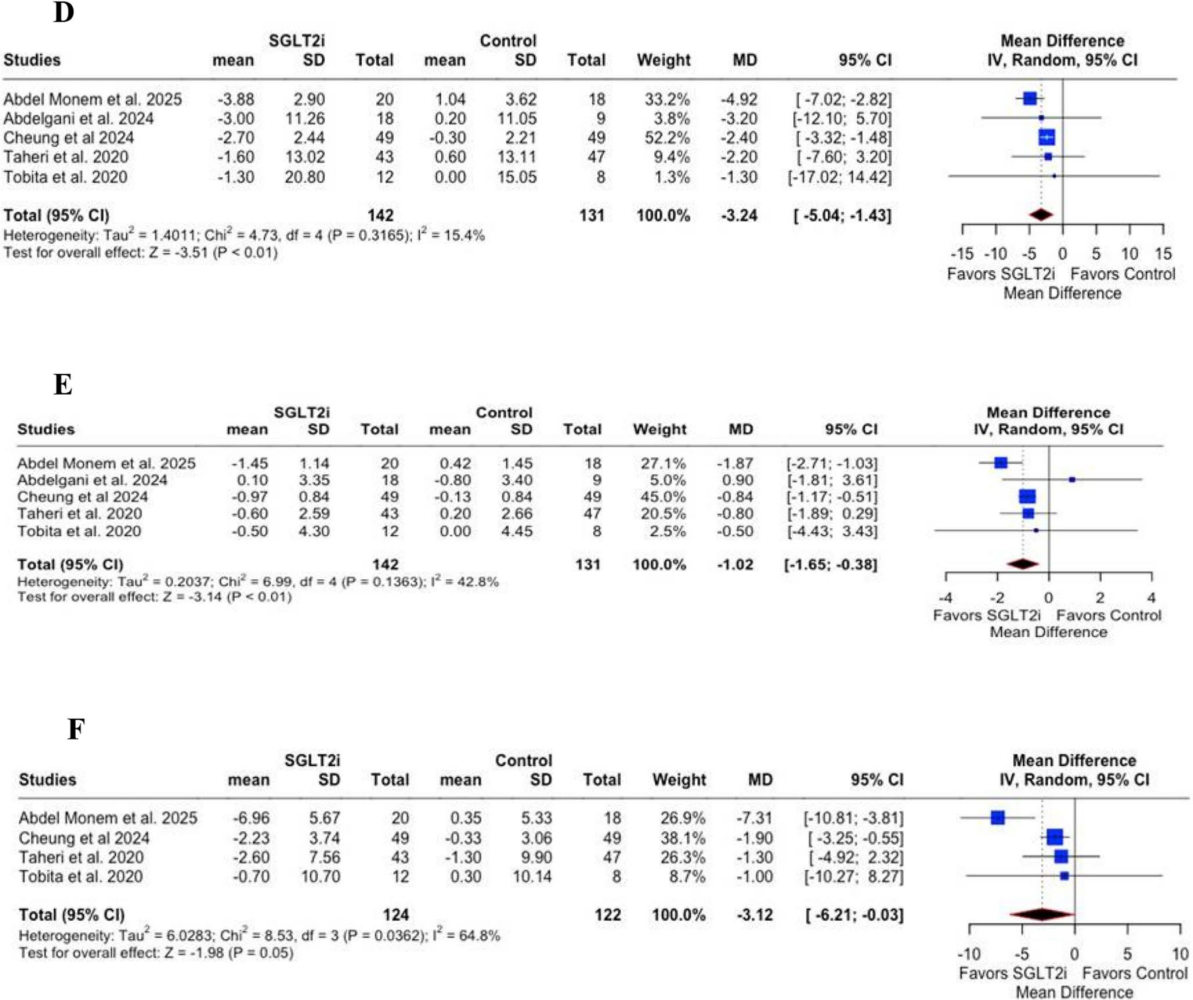
** A** – Triglyceride levels showed no significant difference between the SGLT2i and control groups (*p* = 0.83), **B** – Glycated haemoglobin (HbA1c) showed no significant difference between groups (*p *= 0.53), **C** – Fasting blood glucose (FBG) showed no significant difference between groups (*p* = 0.09), **D** – Body weight was significantly reduced in the SGLT2i group compared with controls (*p* < 0.01), **E** – Body mass index (BMI) was significantly reduced in the SGLT2i group compared with controls (*p *< 0.01), **F** – Waist circumference was borderline significantly reduced in the SGLT2i group compared with controls (*p* = 0.05). CI = Confidence interval; MD = Mean difference

Regarding metabolic parameters, SGLT2inhibitors did not significantly reduce triglyceride levels (MD = −0.80; 95% CI: −8.21 to 6.61; *p* = 0.83; I² = 6.6%; Fig. 3A), glycate hemoglobin (HbA1c) (MD = −0.02; 95% CI: −0.07 to 0.04; *p* = 0.53; I² = 0%; Fig. 3B), or fasting blood glucose (FBG) (MD = −1.54; 95% CI: −3.30 to 0.22; *p* = 0.09; I² = 71.9%; Fig. 3C). However, significant reductions were observed in body weight (MD = −3.24 kg; 95% CI: −5.04 to −1.43; *p* < 0.01; I² = 15.4%; Fig. 3D), body mass index (BMI) (MD = −1.02 kg/m²; 95% CI: −1.65 to −0.38; *p* < 0.01; I² = 42.8%; Fig. 3E), and waist circumference (MD = −3.12 cm; 95% CI: −6.21 to −0.03; *p* = 0.05; I² = 64.8%; Fig. 3F).

### Imaging and fibrosis parameters

**Fig. 4:**
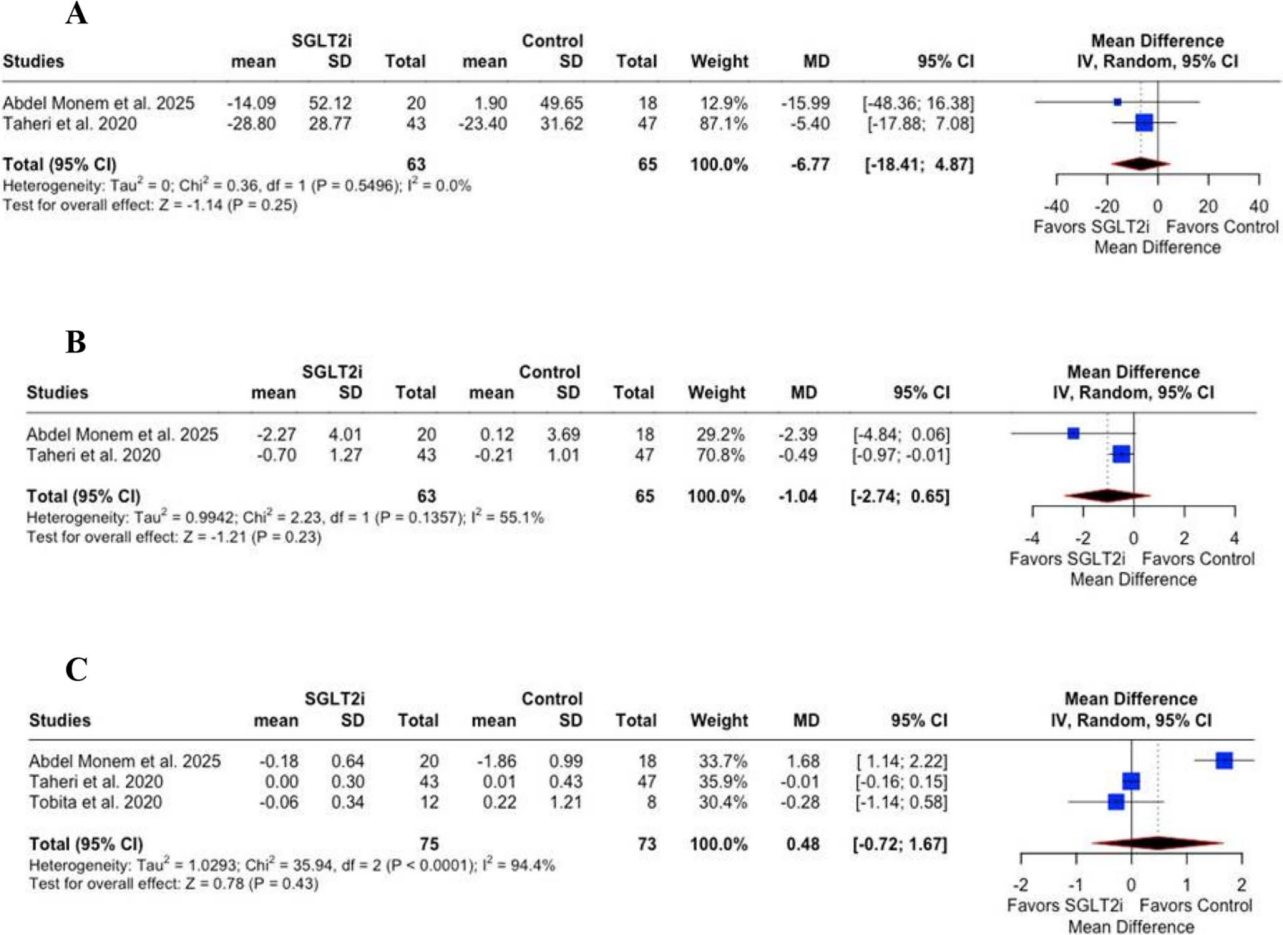
** A **– Controlled Attenuation Parameter (CAP) score showed no significant difference between the SGLT2i and control groups (*p* = 0.25),** B** – Liver stiffness measurement (LSM) showed no significant difference between groups (*p*= 0.23), **C** – Fibrosis-4 (FIB-4) score showed no significant difference between groups (*p* = 0.43). CI = Confidence interval; MD = Mean difference

No statistically significant differences were observed in Controlled Attenuation Parameter (CAP) score (MD = −6.77;95% CI: −18.41 to 4.87; *p* = 0.25; I² = 0%; Fig. 4A), liver stiffness measurement ༈LSM༉(MD = −1.04; 95% CI: −2.74 to 0.65; *p* = 0.23; I² = 55%; Fig. 4B), or Fibrosis-4༈FIB-4༉ score (MD = 0.48; 95% CI: −0.72 to 1.67; *p* = 0.43; I² = 94.4%; Fig. 4C).

### Subgroup analysis

Based on the subgroup analysis, SGLT2i demonstrated a consistent and favorable impact on metabolic and hepatic outcomes compared with other antidiabetic agents, such as pioglitazone and teneligliptin. In terms of liver enzyme reduction, the SGLT2i group showed greater reductions in ALT (MD − 4.50; 95% CI: −6.89 to − 2.10, Supplementary Figure S1.1) and AST (MD − 2.03; 95% CI: −3.24 to − 0.82, Supplementary Figure S1.2) compared to controls, with no statistical difference between subgroups. In BMI reduction, SGLT2i demonstrated a more pronounced effect (MD − 1.02; 95% CI: −1.65 to − 0.38, Supplementary Figure S1.3), with a significant subgroup difference (*P* = 0.0256), indicating superiority over other antidiabetics. Although FBG (Supplementary Figure S1.4) and GGT (Supplementary Figure S1.5) reductions favored SGLT2i, the results were not statistically significant across all comparisons. Notably, GGT showed a trend favoring SGLT2i with a borderline p-value (*P* = 0.06 in the anti-diabetic drug subgroup), while FBG reduction lacked statistical significance.

Furthermore, weight loss and waist circumference reduction were significantly greater in the SGLT2i group (weight MD − 3.24 kg; 95% CI: −5.04 to − 1.43, Supplementary Figure S1.6 and waist circumference MD − 3.12 cm; 95% CI: −6.21 to − 0.03, Supplementary Figure S1.7), supporting their beneficial role in obesity-related metabolic derangements. HbA1c changes (Supplementary Figure S1.8) and triglyceride levels (Supplementary Figure S1.9) showed no significant differences between SGLT2i and other antidiabetic agents, with effect sizes near zero and non-significant p-values, suggesting neutral glycemic control when compared directly. Overall, SGLT2i showed a superior profile in terms of anthropometric outcomes and liver enzyme improvement, with a favorable trend across most markers, particularly in the placebo subgroup, reinforcing their potential role in metabolic dysfunction-associated conditions like MASLD/NASH.

An additional subgroup analysis comparing empagliflozin directly with placebo revealed consistent hepatic and metabolic benefits. Treatment with empagliflozin significantly reduced ALT (MD = − 4.62 U/L; 95% CI − 7.06 to − 2.17; I² = 0%) (Supplementary Fig. 3.1) and AST (MD = − 2.12 U/L; 95% CI − 3.35 to − 0.90; I² = 0%) (Supplementary Fig. 3.2), confirming robust improvement in liver enzyme activity. Significant anthropometric benefits were also observed, with reductions in body weight (MD = − 2.40 kg; 95% CI − 3.31 to − 1.50; I² = 0%) (Supplementary Fig.  3.3), BMI (MD = − 0.81 kg/m²; 95% CI − 1.13 to − 0.50; I² = 0%) (Supplementary Fig. 3.4), and waist circumference (MD = − 1.83 cm; 95% CI − 3.09 to − 0.56; I² = 0%) (Supplementary Fig. 3.5). No significant differences were observed in HbA1c (MD = − 0.02; 95% CI − 0.07 to 0.04; I² = 0%) (Supplementary Fig. 3.6). or fasting blood glucose (MD = − 1.48 mg/dL; 95% CI − 3.45 to 0.49; I² = 84%) (Supplementary Fig. 3.7)., although the direction of effect favored empagliflozin. Collectively, these placebo-controlled findings strengthen the evidence for the hepatic and metabolic efficacy of empagliflozin in non-diabetic MASLD, demonstrating consistent improvements in enzyme levels and weight with minimal heterogeneity across trials.

### Sensitivity analysis

To assess the robustness of our findings, we conducted leave-one-out sensitivity analyses for each primary and secondary outcome. The results demonstrated consistent effect estimates across all analyses, suggesting that no single study disproportionately influenced the overall results. For ALT (Supplementary Figure S2.1) and AST levels (Supplementary Figure S2.2), the exclusion of any individual study did not significantly alter the pooled effect size or confidence intervals, reinforcing the reliability of the observed improvements in liver enzymes. Similarly, sensitivity analysis for body weight (Supplementary Figure S2.3) and BMI (Supplementary Figure S2.4) confirmed the significant reductions, with no changes in statistical significance upon sequential study omission.

Regarding FBG (Supplementary Figure S2.5) and waist circumference (Supplementary Figure S2.6), the sensitivity analysis revealed moderate variability; however, the directionality of effect remained consistent, supporting a trend toward metabolic benefit despite some heterogeneity.

The FIB-4 score analysis (Supplementary Figure S2.7) displayed the most significant variability in effect estimates upon removal of individual studies, consistent with the high baseline heterogeneity in the pooled analysis (I² = 94.4%). This suggests caution in interpreting this outcome and highlights the need for more homogeneous data on liver fibrosis.

The observed heterogeneity in specific outcomes (FIB-4, FBG, and waist circumference) likely reflects clinical diversity among studies, including differences in baseline BMI, intervention duration (12–52 weeks), and comparator arms (placebo vs. active agents). Variability in imaging modality (MRE vs. FibroScan) may have also contributed. Notably, the direction of effect remained consistent, supporting the overall stability of the result.

Egger’s regression test did not identify significant small-study effects for any parameter: waist circumference (intercept = − 0.84, *p* = 0.68), fasting blood glucose (intercept = − 1.26, *p* = 0.25), ALT (intercept = 0.59, *p* = 0.41), AST (intercept = 0.70, *p* = 0.31), Hb1ac (intercept = − 0.27, *p* = 0.66) and weight change (intercept = − 0.34, *p* = 0.69). These results indicate no evidence of significant publication bias (Supplementary Fig. 4.1 to 4.6).

However, given the small number of included studies (k = 5), the statistical power of Egger’s test is limited, as meta-analyses with fewer than ten studies may yield imprecise estimates [26]. Complementary analysis using a Doi plot further confirmed the absence of major asymmetry in effect distribution.

Overall, the sensitivity analyses affirm the stability of the meta-analysis results, particularly for liver function parameters and anthropometric outcomes, and the certainty of evidence for ALT/AST improvement was moderate per GRADE framework [26]. (Supplementary Table 2)

### Quality assessment

**Fig. 5:**
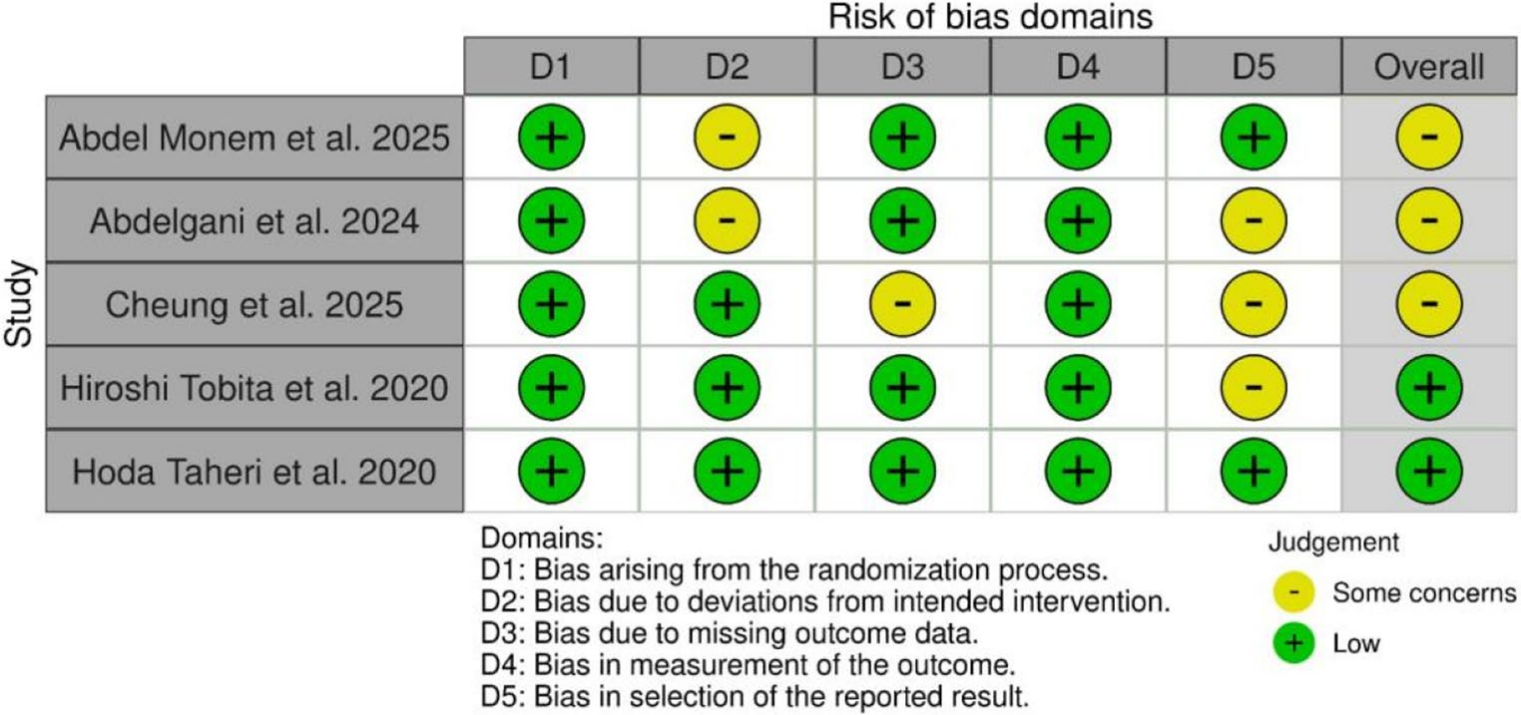
This data is mandatory, please provide

The risk of bias (RoB) assessment using the Cochrane RoB 2 tool revealed an overall low risk of bias in two studies (Hiroshi Tobita et al. 2020 and Hoda Taheri et al. 2020) [27, 28], while the remaining three studies (Abdel Monem et al. 2025, Abdelgani et al. 2024, and Cheung et al. 2025) [29–31] presented some concerns (Fig. 5). All studies showed low risk in randomization and outcome measurement, indicating adequate random sequence generation and objective assessment methods. However, concerns were noted in deviations from intended interventions in two studies and the selection of the reported results in three studies, suggesting possible issues with blinding or protocol adherence and selective reporting. One study (Cheung et al. 2025) [31] raised some concerns due to missing outcome data. Despite these concerns, the overall methodological quality was acceptable, with no study judged to be at high risk of bias in any domain.

## Discussion

Our meta-analysis demonstrated that SGLT2i significantly improved liver enzymes in patients with NASH or hepatic steatosis, with notable reductions in AST, ALT, and GGT levels. While no significant changes were observed in triglycerides, HbA1c, or FBG, there was a meaningful reduction in body weight, BMI, and waist circumference, indicating favorable metabolic and anthropometric effects. Imaging-based and fibrosis-related outcomes, including CAP, LSM, and FIB-4 scores, did not show significant improvement. These findings suggest that SGLT2i may provide hepatic and metabolic benefits, particularly in liver enzyme normalization and weight reduction, although their impact on liver fibrosis and glycemic control in non-diabetic individuals remains inconclusive and warrants further investigation.

NAFLD and MASLD are both liver conditions marked by fat buildup in the liver. NAFLD is diagnosed by exclusion, requiring the presence of hepatic steatosis without significant alcohol intake or other secondary causes such as viral hepatitis or drug-induced liver injury [15, 32]. In contrast, MASLD, a newer term introduced in 2023, is diagnosed by inclusion: it requires liver steatosis plus at least one cardiometabolic risk factor (e.g., obesity, diabetes, hypertension, dyslipidemia, or insulin resistance) [33]. The shift from NAFLD to MASLD aims to better reflect the metabolic causes of the disease and reduce stigma by moving away from the “non-alcoholic” label. As a result, most patients formerly classified as having NAFLD now meet the MASLD diagnostic criteria [33, 34].

NAFLD or MASLD is a highly prevalent chronic liver condition characterized by excessive hepatic fat accumulation. Affecting approximately 25–30% of the global adult population and nearly 30% in the United States [35, 36]. The burden of MASLD is not only clinical but also economic: in the United States alone, the annual direct medical costs associated with NAFLD/MASLD are estimated to exceed $100 billion, with global costs projected to reach hundreds of billions due to complications like NASH, cirrhosis, hepatocellular carcinoma, and cardiovascular disease [37, 38]. As the prevalence of MASLD continues to rise alongside obesity and diabetes, it poses a growing challenge to healthcare systems worldwide.

SGLT2i act by selectively blocking the sodium-glucose co-transporter 2 in the proximal renal tubules, thereby reducing glucose and sodium reabsorption and promoting glycosuria and natriuresis. This results in osmotic diuresis, blood pressure reduction, and modest weight loss, all without increasing hypoglycemia risk in non-diabetic individuals [39, 40]. Beyond glycemic control, SGLT2i have demonstrated significant cardiorenal benefits, attributed to reductions in intraglomerular pressure, attenuation of sympathetic tone, and improvement in endothelial function [41]. They also reduce albuminuria and slow eGFR decline in both diabetic and non-diabetic chronic kidney disease (CKD) patients [42]. Cardiovascular benefits are linked to improved myocardial metabolism (via ketone body utilization), reduction in cardiac preload and afterload, and antifibrotic and anti-inflammatory actions [43, 44].

SGLT2i also exerts favorable hepatic effects in patients with MASLD/NASH, especially in those without diabetes. Mechanistically, these benefits stem from reductions in insulin resistance, visceral adiposity, and systemic inflammation, all of which are key contributors to hepatic steatosis and fibrosis [45, 46]. Clinical studies have shown that SGLT2i reduce liver fat content, lower ALT and AST levels, and may improve histological markers of NASH [15]. Moreover, by lowering hepatic lipogenesis and enhancing beta-oxidation, SGLT2i improves liver metabolic flexibility [47, 48]. In non-diabetic MASLD populations, where pharmacologic options remain limited, SGLT2i provide a promising intervention with dual hepatic and cardiometabolic benefits [49]. As such, they are gaining attention for their role in addressing the pathophysiological overlap between metabolic syndrome, fatty liver disease, and cardiovascular morbidity.

The lack of significant improvement in steatosis and fibrosis parameters (CAP, LSM, FIB-4) may be attributed to the short treatment durations (12–52 weeks) and the limited sample sizes across available trials. Fibrotic remodeling is a slow process, often requiring ≥ 48 weeks (about 11 months) of therapy to yield measurable reductions in stiffness or histologic change [35]. Moreover, variability in imaging techniques and cutoff thresholds for fibrosis staging could have attenuated pooled effect estimates. Future studies with standardized endpoints and histologic confirmation are needed to clarify whether SGLT2i confer antifibrotic benefits. Given the growing global burden of MASLD and its progression to more severe liver disease and hepatocellular carcinoma, the exploration of novel pharmacotherapies remains an urgent priority [50]. Our meta-analysis underscores the hepatic and metabolic benefits of SGLT2i, particularly in improving liver enzymes and reducing body weight, which are crucial in mitigating disease progression [51]. However, the lack of significant improvement in fibrosis-related outcomes highlights the need for more targeted therapeutic strategies and long-term studies [36, 46]. The increasing prevalence of MASLD, compounded by its association with cardiovascular disease and substantial economic impact, calls for a coordinated research effort to develop effective treatments that go beyond symptom management to modify disease trajectory [52]. Advancing our understanding of SGLT2i and similar agents could open new avenues not only for liver-specific outcomes but also for reducing the incidence of cirrhosis, hepatocellular carcinoma, and the broader cardiometabolic burden [53, 54]. Future research should focus on large-scale, randomized trials in diverse populations, including non-diabetic patients, to validate these findings and ultimately guide clinical practice and policy [55].

### Strengths and limitations

This meta-analysis is the first to specifically synthesize RCT data, evaluating the efficacy of SGLT2i in non-diabetic individuals with NAFLD/MASLD, addressing a growing and understudied population. The methodology followed PRISMA 2020 guidelines and was prospectively registered in PROSPERO, ensuring transparency and reproducibility. Key strengths include the exclusive inclusion of RCTs, comprehensive literature search across multiple databases, and robust statistical methods, including sensitivity and subgroup analyses, to evaluate the consistency of findings. Notably, significant improvements were consistently observed in liver enzymes, and anthropometric measures, suggesting a potential therapeutic benefit of SGLT2i beyond glycemic control.

Despite its strengths, this meta-analysis has several limitations. First, the total number of included RCTs and participants was limited (*n* = 5 studies, 273 patients), which may reduce statistical power and generalizability. Second, there was heterogeneity in comparator arms (placebo vs. other pharmacological agents), treatment duration, and outcome definitions across studies. Third, the absence of liver biopsy endpoints and reliance on non-invasive imaging and serum biomarkers limits the ability to definitively assess histological improvement or fibrosis regression. Additionally, high heterogeneity was noted in some secondary outcomes, such as FIB-4 score and FBG, and some included studies presented “some concerns” in risk of bias assessment, particularly regarding outcome reporting and missing data. Finally, the short follow-up durations in available RCTs limit conclusions on long-term hepatic outcomes and safety.

## Conclusion

This meta-analysis demonstrates that SGLT2i provide significant benefits in patients with NAFLD/MASLD, without T2DM, by improving key metabolic and hepatic parameters. These effects were generally consistent regardless of whether SGLT2i were compared to placebo or other antidiabetic agents such as pioglitazone and tenegliptin, although placebo-controlled trials more frequently achieved statistical significance across outcomes. No meaningful differences in certain fibrosis-related parameters, underscoring the need for longer-term studies with histologic endpoints. Collectively, these findings highlight the potential role of SGLT2i as a multifaceted therapeutic option in NAFLD/MASLD management, addressing both metabolic and hepatic disease components, while reinforcing the importance of continued investigation into their long-term impact on liver fibrosis progression and cardiovascular outcomes.

## Supplementary Information

Below is the link to the electronic supplementary material.

Supplementary File 1 (DOCX 4.75 MB)

## Data Availability

The data used in this meta-analysis were extracted from publicly available sources, including published randomized controlled trials. All relevant data supporting the findings of this study are included within the manuscript and supplementary materials. Due to the nature of this analysis, no new primary data was generated. Additional details can be made available upon reasonable request to the corresponding author.

## Abbreviations

AASLD: American Association for the Study of Liver Diseases
ALT: Alanine aminotransferase
AST: Aspartate aminotransferase
BMI: Body mass index
CAP: Controlled attenuation parameter
CI: Confidence intervals
CKD: chronic kidney disease
CVD: cardiovascular disease
ELF: Enhanced liver fibrosis (test)
FIB: 4 score–Fibrosis–4 index
GGT: Gamma–glutamyl transferase
LSM: Liver stiffness measurement
MASLD: Metabolic dysfunction–associated steatotic liver disease
MDs: Mean differences
MRE: Magnetic resonance elastography
NAFLD: Nonalcoholic fatty liver disease
NFS: NAFLD fibrosis score
RCTs: Randomized controlled trials
SGLT2: Sodium–glucose cotransporter–2
SGLT2i: Sodium–glucose cotransporter–2 inhibitors
T2DM: Type 2 diabetes mellitus
TGL: Triglycerides
WC: Waist circumference
